# Preliminary Cleaning Approach with Alginate and Konjac Glucomannan Polysaccharide Gel for the Surfaces of East Asian and Western String Musical Instruments

**DOI:** 10.3390/ma15031100

**Published:** 2022-01-30

**Authors:** Chaehoon Lee, Francesca Volpi, Giacomo Fiocco, Maduka L. Weththimuni, Maurizio Licchelli, Marco Malagodi

**Affiliations:** 1 Department of Chemistry, University of Pavia, 27100 Pavia, Italy; chaehoon.lee01@universitadipavia.it (C.L.); madukalankani.weththimuni@unipv.it (M.L.W.); maurizio.licchelli@unipv.it (M.L.); 2 Arvedi Laboratory of Non-Invasive Diagnostics, CISRiC, University of Pavia, 26100 Cremona, Italy; francesca.volpi@unipv.it; 3 Department of Musicology and Cultural Heritage, University of Pavia, 26100 Cremona, Italy

**Keywords:** sodium alginate, konjac glucomannan, string musical instruments, noninvasive analysis, gel cleaning, hydrogel

## Abstract

The cleaning of string musical instruments is challenging due to the traditional finishing treatments used by the makers. Multilayered coating systems were applied to Western musical instruments, while the Nakdong technique was applied in East Asia. Furthermore, by restorations and performance, dust and grime were overlapped together with polishes, adhesives, and varnishes. Gel cleaning is important in the field of conservation because of the ability to selectively remove chemical and biological degradation products from the surface, minimizing the interactions with the inner layers. In this study, hydrogels based on sodium alginate (SA) and konjac glucomannan (KG) polysaccharides were applied on laboratory mock-ups of East Asian and Western instruments to test their ability to remove synthetic soiling and sweat from the surface. In particular, SA cross-linked with calcium cations and KG cross-linked with borate gels were used. To control the exposure of the cleaning solvent on the surface of mock-ups, the moisture content of the gels was determined. The effectiveness of removing synthetic contaminants was investigated by noninvasive analytical methods. Stereomicroscopy and colorimetry, together with Fourier Transform Infrared (FTIR) spectroscopy in reflection mode and X-Ray Fluorescence (XRF), were used to evaluate the cleaning efficacy. Overall, polysaccharide hydrogels resulted in promising cleaning systems on both smooth and rough surfaces of wood.

## 1. Introduction

In the field of cultural heritage conservation, the cleaning of artworks has become an essential practice, including the removal of substances deriving from natural pollution (e.g., surface dirt, grime, and soil) as well as past nonoriginal treatments (e.g., polishing treatments, adhesives, and varnishes) [1]. Depending on the material and the cleaning purpose, the method has varied from mechanical (e.g., vacuuming or using traditional tools such as brushes, cotton swabs, sponges, and scalpels) to water-based or diluted solvent-based cleanings [2]. Since Wolbers introduced solvent-based gels in the mid-1980s [3,4], new cleaning systems have been tested to remove dirt and degraded materials from metals, stones, and paintings and in the practice of paper deacidification [5,6,7,8]. The gels have differed from physical gels to chemical gels: physical ones are gel-like networks, built by molecules cross-linked through noncovalent interactions; on the contrary, chemical gels are networks cross-linked with covalent bonds. The drawback associated with physical gels is that they may leave solid residues on the treated surfaces [9], and chemical gels rely on the reduction in the spreading of the solvent onto or into the artwork surface, which in turn reduces swelling and leaching issues [10]. 

In the specific field of musical instruments, less effort was devoted to deepening a systematic study on cleanings and their interaction with the materials of musical instruments. Cleaning practices have been mainly developed in the workshops of the master luthiers or restorers, and only a few scientific studies have been dedicated to this issue [11,12]. Musical instruments, and in particular the stringed ones, were handled and played a lot throughout their history, and over the centuries, human dirt has settled on the surfaces. In addition, altered materials and overlapped layers were often present due to many maintenance and restoration procedures carried out on instruments [13,14]. Another cleaning issue is related to the wood, which is subjected to soaking and ultimately swells in the presence of a solvent (including water); solvent cleaning may cause both swelling and leaching of the components, damaging the surface and the layer underneath. For these reasons, when cleaning the surface of historical musical instruments, all the layers need to be preserved, and thus the cleaning needs to be confined to the dirt areas, avoiding interaction with the multilayered system (e.g., original varnishes, pigmented layers, and wooden substrate). 

By paying attention to the sensitivity of historical musical instrument materials, in this research, we would like to introduce two different hydrogels based on sodium alginate and konjac glucomannan polysaccharide, as novel cleaning systems for wooden objects. Particular attention has been drawn to the polysaccharides, such as agar, gellan gum, xanthan gum, and chitosan, as the hydrogels based on natural polymers have been considered as cleaning tools by conservators over the last few decades [15]. Hydrogels contain hydrophilic functional groups and can load water-based cleaning systems. In addition, as natural polymers, they are nontoxic, biologically compatible, biodegradable, and easily obtainable. 

**Figure 1:**
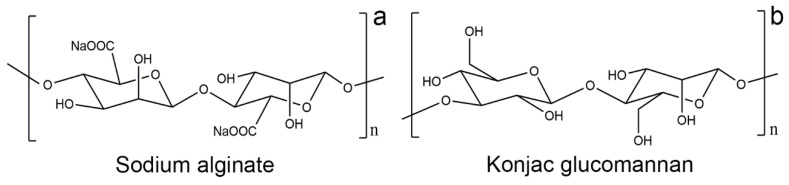
Chemical structure of (**a**) sodium alginate and (**b**) konjac glucomannan.

Alginate is an unbranched polysaccharide extracted from brown algae. It consists of copolymers of β-D-mannuronic acid and α-L-guluronic acid units linked together by 1,4 glycosidic bonds (Figure 1a) [16]. The main form of alginate hydrogel is based on ionic cross-linking with multivalent cations, Ba^2+^, Sr^2+^, Ca^2+^, Mg^2+^, and Al^3+^ [17,18,19]. Due to their interaction with metal cations and their biocompatibility and degradability properties, sodium alginate based hydrogels have been used as adsorbents in wastewater treatment [20], as well as in the food industry and pharmaceuticals [21,22,23,24]. Recently, sodium alginate and polyvinyl chloride gel mixed with an ion exchanger, layered double hydroxides, and α-zirconium phosphate have been used to remove gypsum efflorescence in fresco paintings [25,26].

Konjac glucomannan is a water-soluble neutral polysaccharide derived from konjac tubers; it consists of D-mannose and D-glucose monomers linked by β-1,4 bonds and has a short side branch at the C-3 position of mannose (Figure 1b) [27,28]. The gel is formed by alkaline processing, borate cross-linking, and gelation with other polymers and metal ions [29,30,31,32,33,34]. In addition, it has characteristics such as compatibility with other polymers, biodegradability, water absorption, and retention, so it is widely used also in typical oriental food and in pharmaceuticals and cosmetics [35,36,37,38,39].

Since the study targets historical musical instruments, a set of mock-ups inspired by Western and East Asian string instruments were prepared on the basis of historical and scientific specific literature. As regards the classical bowed string quartet, a number of studies [40,41,42,43,44] often revealed the presence of a multilayered coating system spread on the wooden substrate, composed by a proteinaceous ground layer such as casein or animal glue with an inorganic fraction dispersed therein (e.g., silicates, sulfates, and carbonates), covered by an external varnish layer mostly based on siccative oil mixed with natural resins. As for East Asian musical instruments (e.g., gayageum in South Korea, koto in Japan, and guzheng in China), inorganic or organic pigment mixed with animal glue; lacquer; or typical heat treatment, such as the Nakdong technique, was used. The Nakdong technique is a scorching method applied to the surface of the paulownia wood by using a hot iron. It is conventionally used in most East Asian string instruments to protect against humidity and improve sound quality [45,46]. 

Both the Western and Eastern mock-ups were covered with a dispersion of synthetic soiling or sweat on the surface. After the cleaning procedure with gel application, a preliminary analytical evaluation of the cleaning was performed using noninvasive techniques. In order to observe the difference before and after cleaning, stereomicroscopy and colorimetry were initially used, while Fourier transform infrared (FTIR) spectroscopy in reflection mode and X-ray fluorescence (XRF) were applied to provide the primary removal state of organic and inorganic substances on the surface. The aims of this study are (i) to introduce two novel water-based cleaning systems suitable for wooden substrates in the field of musical instruments and (ii) to provide preliminary observations of the interaction between the new hydrogels and the Western and East Asian finishing surfaces. 

## 2. Materials and Methods

### 2.1. Mock-Up Preparation

**Figure 2:**
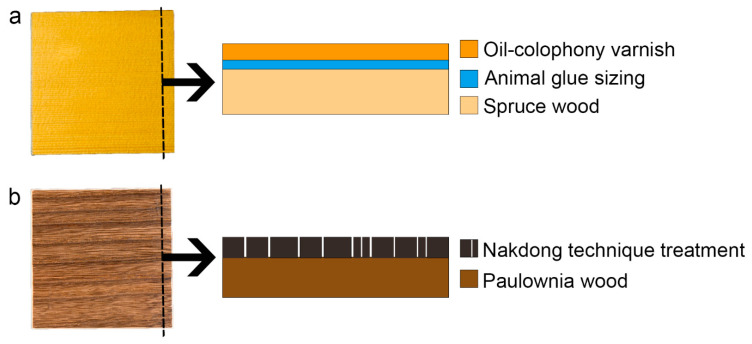
Schematic reconstruction of wooden mock-ups representative of (**a**) Western and (**b**) East Asian musical instrument finishing treatments.

Spruce, used for the Western mock-ups (WMs), and paulownia woods, used for East Asian mock-ups (EAM), were cut in 10 cm × 10 cm × 1 cm (longitudinal × radial × tangential direction). To prepare WMs, a rabbit glue (10% *w*/*w* water solution) ground layer was applied using a brush. After that, two applications of oil-based varnish, composed of linseed oil and colophony (1:1 ratio), were applied by brush (Figure 2) [42]. After applying the varnish, all samples were dried under UV light for 30 h. To prepare EAMs, the Nakdong technique was applied. A traditional iron tool specifically used for this treatment, customized by a blacksmith, was heated inside the hot kiln stove until it reached up to 1000 °C. After that, the iron tool was pressed with one stroke for 1-3 s along the direction of the wood grain. Lastly, all of each burnt surface was brushed off by brush [46].

### 2.2. Contaminant Deposition

The dry portion of the synthetic soiling mixture consisted of the following ratios (w/w): iron oxide (burnt sienna pigment, 0.5%), gelatin (10.4%), soluble starch (10.4%), Portland type I cement (20.5%), silica (1.9%), lime (16.7%), kaolin (18.6%), and peat moss (20.8%); it was made by minimally changing the original soiling mixture of Wolbers [3,47]. The wet portion was prepared with mineral oil in chloroform (4.5% *v*/*v*). All the soiling components were purchased from Bresciani S.R.L., Milan, Italy [11]. In the case of synthetic soiling, 1.1 g of dry portion and 20 mL of wet portion were mixed. By using an airbrush at a 20 cm distance, the soiling mixture was evenly dispersed on the East Asian and Western mock-up samples. The synthetic sweat, which mimicked human hand sweat at pH 2.3 according to DIN ISO 9022-12, was purchased from Synthetic Urine e.K. (Eberdingen-Nussdorf, Germany). All samples were sprayed with synthetic sweat by repeating the spraying and drying process five times in order to simulate the contact situation of the instrument user.

### 2.3. Preparation of Polysaccharide Cleaning Hydrogels

For the sodium alginate (SA) gel system, a medium viscosity (A2033), Mw ~80,000 to 120,000 g/mol, gel was purchased from Sigma Aldrich (Saint Louis, MO, USA). For the exploration of SA, the one-pot method was performed with various multivalent cations (Ba^2+^, Sr^2+^, Ca^2+^, Mg^2+^, and Al^3+^), using different concentrations (1%, 2%, 4% *w*/*v*, in distilled water) and reaction times (5, 15, and 30 min) to find a good texture for cleaning purposes. The pH of the gels was measured using a paper strip (Carlo Erba, Italy), and the gelation characteristics are presented in Table S1 and Figure S1 in Supplementary Information. SA gel was prepared with deionized water by stirring at room temperature until completely dispersed. The SA solution was poured into a petri dish until it reached a thickness of 2-3 mm. This should be done on a flat area to make an even film shape. In the prepared container, CaCl_2_ 2% (*w*/*v*) solution in distilled water was poured to immerse the SA solution for the reaction in a molar ratio of 1:900 for 15 min. The prepared SA cleaning system was gently rinsed with deionized water and immersed in the 2% of Ecosurf EH-9 (EH-9, Sigma Aldrich, Saint Louis, MO, USA) solution (*v*/*v*, in distilled water) for 24 h to make it ready for use. EH-9 is known as a nonionic surfactant that is easily biodegradable and effective in removing oily soils [48,49]. 

For the konjac glucomannan (KG) gel system, a purity ≥95%, Mw ~960,000 g/mol gel was provided by Hubei Yizhi Konjac Co. Ltd (Yichang, China). The exploration of KG was carried out with a one-pot method using various alkali chemicals NaOH, KOH, and Ca(OH)_2_ with different concentrations of KG (1%, 2%, 4% *w*/*v*, in distilled water). Different concentrations of borax were also tested for two cis-diol pairs of KG and borate ion interchain cross-linking. KG gel was prepared at 50 °C, and while stirring, 0.1 g of borax was added in 10 mL KG solution (Molar ratio 1:6250). The KG solution was left at room temperature for 30 min to stabilize the mixture. Thereafter, the stabilized mixture was placed in bulk form in deionized water, maintaining the temperature at 90 ± 10 °C for 20 min. The immersed KG gel was taken out, cooled, and dried at room temperature until the water on the surface dried. The prepared KG cleaning system was immersed in 2% of EH-9 solution (*v*/*v*, in distilled water) for 5 h and was then ready for use. 

### 2.4. Cleaning Test 

**Figure 3:**
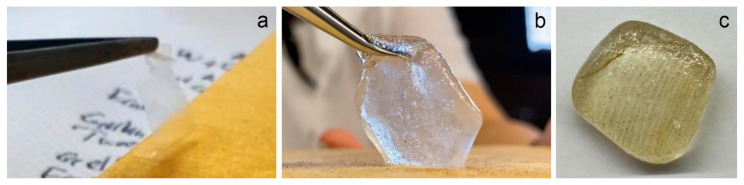
Images related to the application of (**a**) SA gel on the sweat-Western Mock-ups (WM) surface and (**b**) KG gel on the soiled-WM surface; (**c**) surface of KG gel after cleaning the soiled-East Asian Mock-ups (EAM).

Among the prepared gels, SA 2% (*w*/*v* in distilled water) immersed in CaCl_2_ 2% (*w*/*v*) solution for 15 min and KG 2% (*w*/*v* in distilled water) with borax were selected for cleaning tests as the most promising systems. Each gel was loaded with EH-9 (2% *v*/*v* in distilled water), and the cleaning was carried out for 5 min each time; gel application was repeated up to three times (SA_1, SA_2, SA_3, KG_1, KG_2, KG_3) on surfaces on which synthetic soil and sweat were dispersed (Figure 3). Both gels were applied and tested within a week with no significant differences in their performances. For comparison, the gel itself (SA_0, KG_0) was also assessed to confirm whether it could be used as a cleaning system without the surfactant (EH-9).

### 2.5. Determination of Moisture Properties of the Gels

For the SA and KG gels used for cleaning tests, the moisture properties were measured. Determination of these properties was obtained by calculating the gel content (G), the equilibrium water content (EWC), the swelling capacity (SC), and the retention capability (RC) of the gel. The mass of the dried gel and the mass of the initial mixture were weighed using an analytical balance (ACJ 320-4M, KERN, Balingen, Germany). 

Gel content (G) was used to evaluate the gel fraction. It was calculated by Equation (1) [5,50], where W_d_ is the initial weight of the dried sample (g) and W_0_ is the initial weight of the mixture (g):

Gel content, G (%) = (W_d_/W_0_) × 100 (1)

EWC, which provides information on the hydrophilicity of the polymer network, was calculated Equation (2) [5], where W_w_ is the weight of a sample with absorbed water (g):

Equilibrium water content, EWC (%) = [(W_w_ − W_d_)/W_w_] × 100 (2)

To obtain a fully swollen state, SA gel was completely dried and immersed in water for 7 days to provide the W_w_. For KG gel, the fully swollen state was checked with rhodamine B colorant (Fluka, product No. 83690) by immersing the dried KG gel into the colorant–water solution and checking the dying of the gel. The measurement of the weight of the wet (W_w_) gel was performed after 6 h. 

The swelling capacity represents the percentage of solvent that can be loaded to the gel network [51,52]. It was calculated by Equation (3):

Swelling capacity, SC (%) = [(W_w_ − W_d_)/W_d_] × 100 (3)

The retention capability of the gel provides information on the power of the gel network to retain the solvent or to disperse it on a porous surface [53]. The completely swollen hydrogel was placed on three sheets of filter paper inside a plastic container with a lid to prevent evaporation. The sheets of the filter paper were weighed before and 30 min after gel application. SA and KG gels, 1 × 1 cm^2^ area and 2 mm thickness, were used [5]. RC was calculated by equation (4), where F_d_ is the initial weight of filter paper (g) and F_w_ is the weight of filter paper with delivered moisture from the gel (mg):

Retention Capability, RC (mg/cm^2^) = [(F_w_ − F_d_)/1] × 100 (4)

### 2.6. Noninvasive Analysis

Details of soil and sweat dispersed on the mock-up surfaces were observed in visible and UV light using an Olympus stereomicroscope (SZX10, Olympus, Tokyo, Japan) equipped with an Olympus HD DP73 camera. Images were acquired using Stream Essentials software (ver. 2.1., Olympus, Tokyo, Japan).

Colorimetric measurements were carried out using a portable Konica Minolta spectrophotometer (CM-2600d Konica Minolta, Tokyo, Japan), and L^*^a^*^b^*^ (CIE color space, 1976) values were measured using the SpectraMagic NX software (ver. 3.31., Tokyo, Japan). Since the visual appearance of East Asian and Western mock-up surfaces is different, the average value of the specular component excluded (SCE) mode was used for further calculation. In order to understand the difference between before and after cleaning, △E values were calculated by the following equation:

Chromatic difference, △E^*^ = [△L^*2^ + △a^*2^ + △b^*2^] ½ (5)

XRF measurements were carried out using the EDXRF spectrometer ELIO (Bruker Optics, Billerica, MA, USA), with Rh anode with a 1 mm collimator. Analyses were performed by setting a tube voltage of 40 kV, a tube current of 40 µA, and a measurement time of 480 s. XRF values presented in Section 3.4 correspond to the net area counts of the Kα peak of each element normalized to time and to the average of the entire dataset of net area counts of the coherent scattering Rh-Kα peak [54]. The average value was calculated on at least three measurements with the related standard deviation. XRF mapping was carried out at the same voltage and current settings, with a measurement time set at 3 s and a step size of 1 mm. Data were collected with Elio software (ver. 1.6.0.29, Bruker Optics, Billerica, MA, USA).

Reflection FTIR was carried out with a Bruker Alpha portable spectrometer (Bruker Optics, Billerica, MA, USA). The measurement was performed at a 5 mm distance through the external reflectance module composed of an optical layout of 23°/23°. Spectra were collected between 7500 and 375 cm^−1^ at a resolution of 4 cm^−1^ with an acquisition time of 2 min. Data were acquired and processed with OPUS 7.2 software. When required, the reflection spectrum was transformed to absorbance spectra by Kramers–Kronig (KK) algorithm included in the software package (ver. 7.2, OPUS, Billerica, MA, USA).

## 3. Results and Discussion

### 3.1. Moisture Properties of the Gels 

The moisture properties of the selected gels, obtained as described in Section 2.5, are summarized in Table 1. The KG gel resulted in a gel content value two times greater than that of SA, implying a higher degree of cross-linking of the KG network and thus possibly less residue releasing of KG compared to SA gel. Moreover, the equilibrium water content and the swelling capability of KG are considerably higher than those of SA, suggesting that KG is capable of loading a greater amount of water solvent than SA, which is an important advantage to be considered for cleaning systems. SA shows a lower value of retention capability than KG, implying that a smaller amount of water was released by SA when in contact with an absorptive surface (the filter paper). However, the retention capability value of KG, which is greater than the one related to SA (14.2 ± 1.0 and 5.4 ± 1.0, respectively), could be explained by a large amount of water loaded in the KG gel, as confirmed by the swelling capacity value (1.5 × 10^3^ ± 0.3 × 10^3^%). It is worth mentioning that the ability of the KG gel to retain the solvent in its network, avoiding excessive releasing and spreading of the solvent after cleaning, is of utmost importance for conservation treatment because it is directly linked to the risk of causing long-term degradation of the cleaned surface. Previous studies on the moisture properties of hydrogels commonly used in artwork cleaning (i.e., AgartArt and Kelcogel), calculated using the same method described in our work [5,55], revealed that the retention capability of AgartArt (30 mg/cm^2^) and Kelcogel (33 mg/cm^2^) is two times and six times higher than those of SA and KG, respectively, as reported in Table 1. These results indicated that, overall, the novel SA and KG hydrogels showed promising results of efficient moisture-controlling action compared to traditional hydrogels commonly and widely used for cleaning artistic and historical objects. Among the two gels, KG appeared to have both higher swelling capacity and higher water-solvent absorbance capacity than SA, possibly due to its high hydrophilicity.

### 3.2. Stereomicroscopy Observations

**Figure 4:**
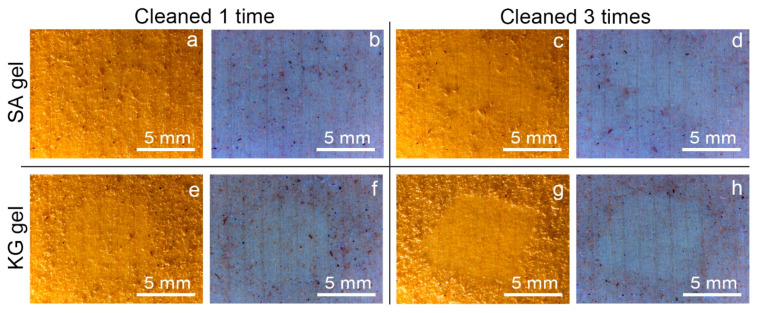
Stereomicroscopy images in visible and UV light of soiled-WM cleaned at different application times with SA and KG gels: (**a**,**b**) SA_1, (**c**,**d**) SA_3, (**e**,**f**) KG_1, and (**g**,**h**) KG_3.

**Figure 5:**
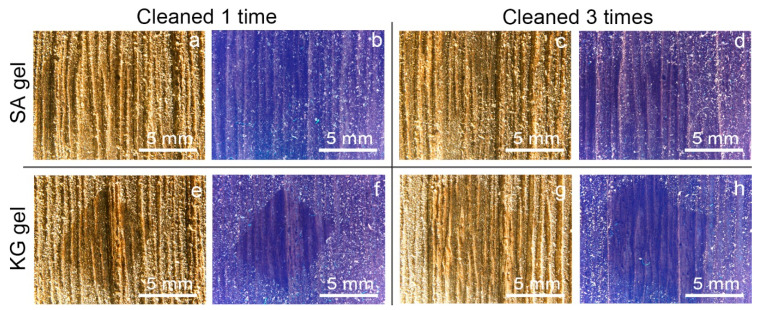
Stereomicroscopy images in visible and UV light of soiled-EAM cleaned at different application times with SA and KG gels: (**a**,**b**) SA_1, (**c**,**d**) SA_3, (**e**,**f**) KG_1, and (**g**,**h**) KG_3.

Macroscopic and microscopic observations of the surfaces of the two kinds of mock-ups allowed us to highlight a considerably different superficial roughness: the WM, covered with varnish, appeared to have a mostly smooth and flat surface; on the contrary, the EAM shows a strong surface roughness with the exposure of ray initial and fusiform initial cells. From the stereomicroscopy observation on the soiled-WM and soiled-EAM after cleaning with SA gel (Figure 4 and Figure 5), significant removal did not appear on SA_1 (Figure 4a,b and Figure 5a,b) and SA_2 treated areas. After repeating the cleaning on SA_3, some soiling particles were unevenly removed, scarcely providing access to observe the smooth surface of the varnish underneath (Figure 4c,d and Figure 5c,d). Compared to the SA, KG gel evidently removed the soil particles already from KG_1, showing an evident outline between the soiled and cleaned area after KG_3 (Figure 4e–h and Figure 5e–h). Furthermore, on soiled-EAM, KG gel seemed to have detached the particles accumulated inside the wood’s ray initial cells, keeping them attached to its surface, as shown in Figure 3c. In addition, comparing the area that was cleaned by the gel without (SA_0 or KG_0) and with EH-9 (SA_1 or KG_1), there was no clear difference between the applications _0 and _1. However, among the two gels, KG_0 showed a minimal increment in soil removal compared to SA_0, showing apparent boundaries of the cleaned area as shown for KG_1 in Figure 5e,f.

After dispersing the sweat on the mock-ups, the sweat marks were only observed on the surface of the WM, while on the EAM, it was hardly recognized by stereomicroscope. This difference could be due to the hydrophobic varnish layer in the WM, which allowed more superficial crystallization of the dried sweat particles than the rough surface of EAM. After cleaning the sweat-WM, the removal efficacy was revealed only by UV light. Both SA and KG gels, with no difference by repeating the cleaning, were shown to partially remove the crystallization of sweat from the surface.

### 3.3. Colorimetric Measurements 

The complete data set with the color coordinates L^*^, a^*^, and b^*^ acquired on both WM and EAM, before and after cleaning, is summarized in Table 2. The overall chromatic variation (△E^*^) is reported in Table 3.

**Figure 6:**
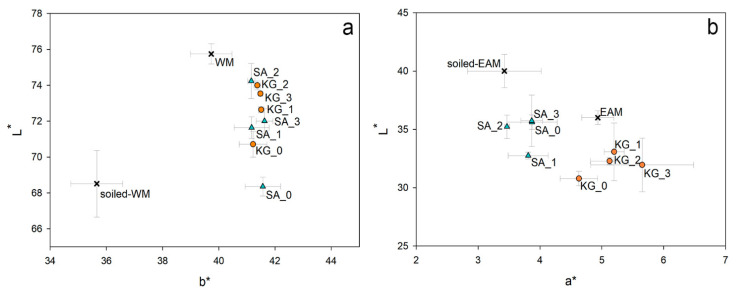
Scatter plots showing (**a**) L^*^ and b^*^ values measured on WM, soiled-WM, and areas repeatedly cleaned with SA and KG gels and (**b**) L^*^ and a^*^ values measured on EAM, soiled-EAM, and areas repeatedly cleaned with SA and KG gels.

On the WM, cleaning of the soiled surface by SA or KG gels highlighted some differences due to the removal efficacy of the two gels. By comparing the L^*^ and b^*^ values collected on the same area before and after cleaning by repeated trials (SA_ and KG_1, 2, and 3), SA_2 and KG_2 show the most considerable difference in L^*^ value (△L = 5.7 and 5.5, respectively). In contrast, all b^*^ values are quite close to the b^*^ value of the surface without the soiling mixture (39.7 ± 0.7) already from the first application (SA_1 and KG_1). SA_0 (△L = −0.2) and KG_0 (△L = 2.2) show the most negligible difference in L^*^, although SA gel seems less effective than KG when no surfactant is added, as previously observed in Section 3.2 in this manuscript. The scatter plot in Figure 6a clearly evidences that by using the surfactant EH-9 and increasing the number of cleaning repetitions from one to three, the ability to remove the soiling mixture is enhanced both for SA and KG gels. At these conditions, indeed, both the brightness and the contribution of the yellow color, respectively indicated by L^*^ and b^*^, measured on the cleaned areas increase to reach values similar to the bright and yellow surface of the WM surface.

On the EAM areas cleaned by SA gel two and three times (SA_2 and SA_3), it was observed that the related L^*^ values (35.2 ± 1.0 and 35.7 ± 2.2, respectively) are close to the L^*^ value of the area without the soiling mixture (36.0 ± 0.6), implying a good removal. Regarding KG applications, L^*^ value shows a gradual decrease by applying KG_1, KG_2, and KG_3 (△L = −6.9, −7.7, and −8.1, respectively). It is worth noting that L^*^ values on EAM decrease from the soiled to non-soiled surface due to the bright color of the synthetic soil compared to the dark wooden surface, and this trend varies in the opposite way for WM. Moreover, the application of KG gel induces a larger variation of the a^*^ parameter than SA gel. The scatter plot of L^*^ and a^*^ values in Figure 5b shows that both SA and KG gels darkened the surface after the cleaning due to the removal of the bright soiling by repeated application times. Overall chromatic variations (△E^*^ in Table 3) indicate that KG gel, in general, affects the color of the EAM surface to a larger extent than SA.

### 3.4. XRF

XRF investigation was aimed to evaluate the effectiveness of cleaning tests through single points and mapping analyses. To reach this point, the elemental composition of synthetic soil and sweat, as well as WM and EAM without any deposition, was characterized, and the marker elements of each material were selected. On WM, the signals of K (Kα = 3.31 keV), Ca (Kα = 3.72 keV), Ti (Kα = 4.52 keV), Cr (Kα = 5.50 keV), Mn (Kα = 5.90 keV), and Zn (Kα = 8.69 keV) were identified as characteristics of the nontreated multilayered coating system. For EAM, signals of K, Ca, Ti, Cr, Mn, and Zn and a weak signal of Fe (Kα = 6.43 keV) were detected. Detected elements, K, Ca, Ti, Cr, Mn, and Zn, on both mock-ups derive mainly from the wood [56,57], although K and Ca increase in count in WM, probably due to the presence of the ground and varnish layers [42,58]. Regarding the soiling mixture applied on WM and EAM, Si (Kα = 1.74 keV), K, Ca, and Fe were the principal elements detected: Ca (kaolin, cement), Fe (cement, burnt sienna pigment), K (peat moss, kaolin), Si (kaolin, silica, cement). In the case of synthetic sweat, only Cl (Kα = 2.64 keV) was recognized due to the organic nature of the mixture, and it was assumed to derive from sodium chloride and ammonium chloride [59].

**Figure 7:**
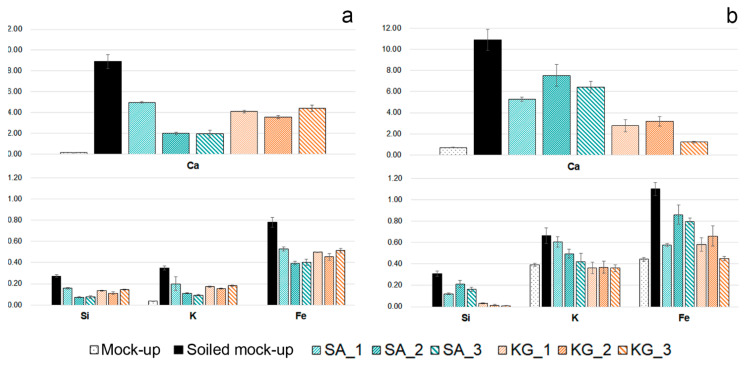
Normalized counts of Ca, Si, K, and Fe detected on (**a**) WM and (**b**) EAM. Different bars correspond to clean mock-ups (WM and EAM); to soiled mock-ups (soiled-WM and soiled-EAM); and to the soiled mock-ups after 1, 2, and 3 applications of SA (SA_1, SA_2, SA_3) and KG gels (KG_1, KG_2, KG_3). XRF values correspond to the net area counts of the Kα peak of each element normalized to time and to the average of the entire dataset of net area counts of the Rh-Kα peak with the related standard deviation.

**Figure 8:**
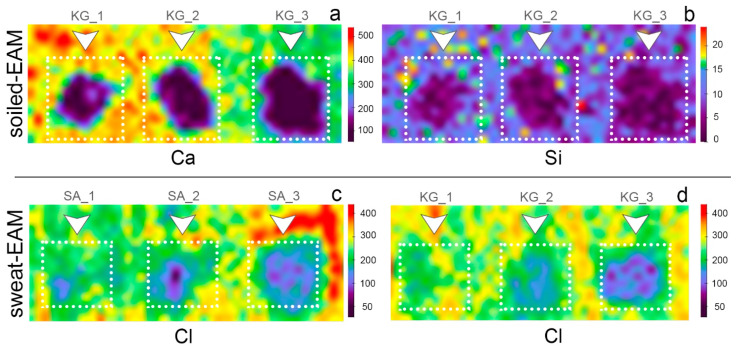
XRF maps of (**a**) Ca (Kα) and (**b**) Si (Kα) on the soiled-EAM after cleaning trials with KG gel (KG_1, KG_2, KG_3, respectively from left to right); XRF map of Cl (Kα) on the sweat-EAM after cleaning trials with (**c**) SA and (**d**) KG gels at different application times (SA_1, SA_2, SA_3, KG_1, KG_2, KG_3, respectively from left to right).

The major compositional changes observed on both soiled-EAM and soiled-WM after cleaning with SA and KG gels were in the Ca, Si, K, and Fe counts. Figure 7 displays the normalized area counts of these peaks before and after the cleaning. A different removal tendency is observed for the two different gels: the removal tendency of SA gel seemed to be increased by repeated trials from SA_1 to SA_3, while KG gel performed an effective removal already from KG_1. When comparing the soiled-WM and SA_3 cleaned areas, element counts progressively decreased to lower values: Ca decreased from 8.90 to 1.94, Fe from 0.78 to 0.40, Si from 0.27 to 0.07, and K from 0.35 to 0.09 (Figure 7a, light blue bars). On the contrary, as shown in Figure 7a (orange bars), KG_1 cleaned area values (Ca 4.11, Fe 0.50, Si 0.13, K 0.17 normalized counts) are comparable with those obtained on the KG_3 areas (Ca 4.44, Fe 0.51, Si 0.14, K 0.18). Moreover, on EAM, KG gels performed a similar cleaning trend, increasing the efficacy of soil removal with repeating trials from 1 to 3 (Figure 7b, orange bars). This trend could be also understood by the XRF elemental maps reported in Figure 8a,b, which indicate a cleaning effectiveness of EAM from KG_1.

When comparing the results achieved on sweat-WM, both SA and KG gels appeared to consistently remove the particles, as suggested by the decrease in Cl counts in Table 4. On sweat-EAM, the removal is increased by an increase in the cleaning trials. Interestingly, the Cl value on the sweat-WM control is higher than the corresponding sweat-EAM value (11.94 and 3.35, respectively), in accordance with the stereomicroscope examinations that showed dried sweat particles (as crystallized drops) on the sweat-WM while they were hardly visible on sweat-EAM. XRF maps of sweat-EAM cleaned areas in Figure 8c,d shows that Cl counts decrease as the trials of cleaning increase, both for SA and KG gels, suggesting a satisfying sweat removal capability.

### 3.5. FTIR Spectroscopy

**Figure 9:**
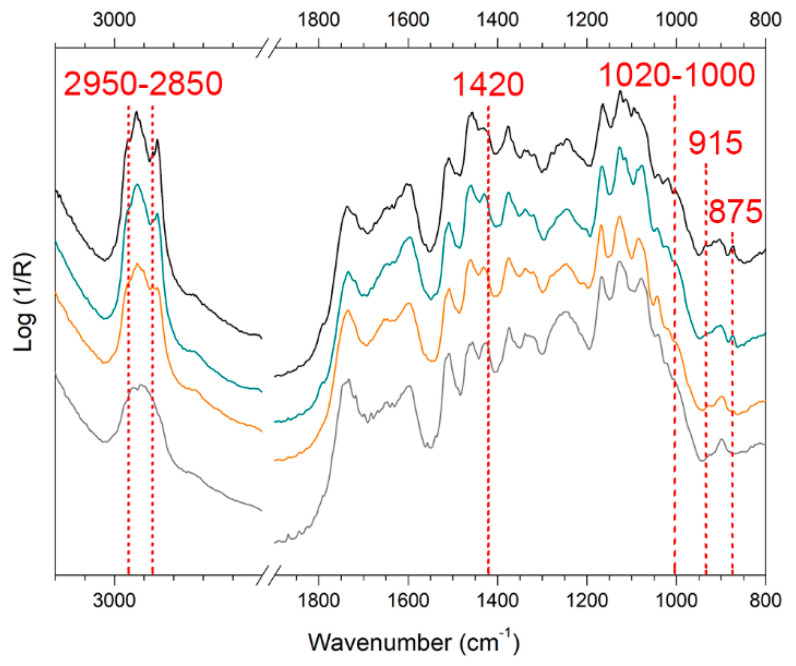
Reflection Fourier Transform Infrared (FTIR) spectra in pseudo-absorbance acquired on soiled-EAM (black), area cleaned with SA_3 (green) and KG_3 (orange) gels, a reference of EAM without soiling mixture (gray). The marker bands selected for identifying soiling mixture and synthetic sweat components are reported.

**Figure 10:**
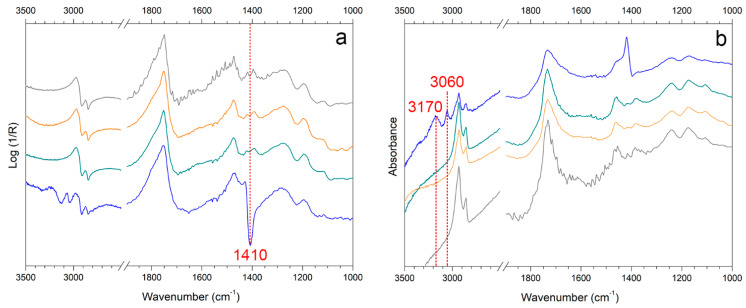
Reflection FTIR spectra in (**a**) pseudo-absorbance and (**b**) after Kramers-Kronig transform of WM (gray) and sweat-WM (blue) cleaned with SA_3 (green) and KG_3 (orange). The marker bands selected for identifying soiling mixture and synthetic sweat components are reported.

As observed for XRF data in Section 3.4, WM and EAM show noticeable different spectroscopic features also by FTIR. All the significant bands identified as markers (both in pseudo-absorbance and KKT) are reported in Table S2. As for EAM, the bands were all studied and interpreted in the pseudo-absorbance spectra as the Nakdong technique caused a very high surface roughness (Figure 5) and a consequent predominant presence of the diffuse contribution [60]. EAM surface without depositions displays the bands related to wood around 1740 cm^−1^ (νC=O, hemicellulose) and at 1595, 1510, 1460, and 1240 cm^−1^ (lignin-related bands) [61,62,63,64,65] (Figure 9). Conversely, in the KK transformed spectra (Figure 10), WM displays the spectral profile of the oil–colophony varnish with characteristic bands around 3000 and 2800 cm^−1^ (νCH_3_ and νCH_2_), around 1735 cm^−1^ (νC=O), at 1460 and 1380 cm^−1^ (δC-H), and at 1250 cm^−1^ and 1170 cm^−1^ (νC-O) [12,63,66]. 

When the soiling mixture was added to EAM (Figure 9, black spectrum) and WM, the pseudo-absorbance spectra showed few characteristic bands of kaolin at 1020–1000 cm^−1^ and 915 cm^−1^ [12,47,55,64,65,66,67]. In addition, there was a contribution from calcium carbonate around 1420 cm^−1^ and at 875 cm^−1^, probably derived from the cement [12,47]. The organic components, such as gelatin, starch, and peat moss, contributed to the bands around 2900 cm^−1^ [12,66,68]. The analysis of sweat showed the inverted band (Reststrahlen) at 1420 cm^−1^ (νC-N) in the pseudo-absorbance spectra (Figure 10a, blue spectrum) and the bands at 3170 and 3060 cm^−1^ (νC-H and νN-H) in the KK transformed spectra (Figure 10b, blue spectrum), which are assumed as the body fluid composition of the mixture of DIN ISO 9022-12 [69,70,71,72].

When comparing the spectra acquired on the areas repeatedly cleaned with the gel, the characteristic bands of WM and EAM progressively appeared, while some bands related to the contaminants slightly decreased, implying that some components of the soiling mixture or sweat were removed. On the surface of soil-EAM (Figure 9), the areas cleaned by repeated trials (KG_3 and SA_3) showed slight variation in the spectral profiles: the bands attributed to kaolin at 1020 cm^−1^ and 915 cm^−1^ and to calcium carbonate at 1430-1420 cm^−1^ decreased in intensity. As for the calcium carbonate, the band at 875 cm^−1^ decreased to the point of disappearing in the KG_3 area (Figure 9, orange spectrum), suggesting a better removal of soiling by KG repeated trials than SA. Similar results were achieved on soil-WM (not showed), where KG gel showed a more efficient cleaning tendency compared to SA. Anyway, both the gels needed to be applied three times before obtaining some detectable spectral changes.

On sweat-WM (Figure 10), all of the cleaning systems, with no distinction of gel or repeated application time, clearly induce the disappearance of the bands related to sweat (Figure 10a,b, blue spectrum). On the contrary, on sweat-EAM, it was difficult to detect the representative peaks of sweat. As already stated for stereomicroscope and XRF results, it was assumed that the sweat deposition might have been absorbed on the rough surface of EAM, which makes its detection challenging. 

## 4. Conclusions

The use of two novel cleaning systems, namely sodium alginate (SA) and konjac glucomannan (KG) hydrogels, applied on the surface of Western and East Asian historical musical instrument mock-ups to remove dispersed soil and sweat was preliminarily investigated and documented through noninvasive analytical methods. Stereomicroscopy and colorimetry were used to observe differences before and after cleaning, while Fourier transform infrared (FTIR) in reflection mode and X-ray fluorescence (XRF) spectroscopies were applied to preliminarily evaluate the removal of organic and inorganic substances from the mock-up surface. Stereomicroscopic observations (Figure 4 and Figure 5) and colorimetric measurements (Figure 6) on both soil-WM and soil-EAM, after cleaning with SA and KG gel, highlight that SA seems not as efficient as KG gel in the removal of soiling mixture. On the contrary, XRF performed on the areas cleaned by SA gel (Figure 7, light blue bars) clearly highlights a decrease in counts of the soil-marker elements such as Ca (kaolin, cement), Fe (burnt sienna pigment, cement), and Si (kaolin, silica, cement). FTIR spectra acquired on the same areas (Figure 9) show a slight decrease in intensity of the marker bands attributed to the silicates (kaolin, cement, burnt sienna pigment) and organic components (peat moss and gelatin), suggesting an action, although partial, of soil removal. Additionally, XRF results suggested that by the increase in cleaning application time (SA_1 to SA_3 and KG_1 to KG_3), the removal of soiling mixture increased on both WM and EAM, as visible in Figure 8a,b.

The sweat-WM presented a slightly different situation on the surface, since sweat was not absorbed into the wood, but instead, it seemingly displayed a crystalline state. As a consequence, it was removed easily with SA and KG gel, as confirmed by the preliminary observation through stereomicroscopy and XRF investigation that showed an evident decrease in counts of Cl, as a marker of sweat (Table 4). For the sweat-EAM, the gels also showed their ability to remove the deposited sweat, as verified by both XRF single point and mapping (Figure 8c,d) that show a progressive decrease in counts of Cl and confirm the cleaning efficacy by repeating the cleaning trials. When comparing SA and KG gels, the second appeared to have a higher removal tendency of sweat than SA gel, as is expected by the result of efficient swelling capacity and high hydrophilicity. To conclude, the preliminary trial results of two novel polysaccharide gels, applied for the first time in the field of Western and East Asian musical instruments, suggest that they are promising materials to approach the cleaning of sensitive surfaces such as treated or varnished wood. However, the rheological properties of the gels and their stability, as well as testing different cleaning solvent trials, were not addressed in this study, but they will be addressed in further research. Moreover, the noninvasive campaign confirmed the need to deepen the results through further quantitative analyses and surface investigation aimed at verifying both the residues of soiling and sweat and the residues arising from the gel at the micro- and nanoscale.

## Figures and Tables

**Table 1:** Moisture properties of SA and KG gels used for the cleaning test, namely gel content (G), equilibrium moisture content (EWC), swelling capacity (SC), and retention capability (RC). The average values and the related standard deviations were determined by repeating the experiment five times. D.W. = distilled water.

Gel	Used Solvent	G (%)	EWC (%)	SC (%)	RC (mg/cm^2^)
SA	D.W.	43 (±5)	64 (±5)	(1.8 ± 0.4) × 10^2^	5.4 (±1.0)
KG	D.W.	85.2 (±0.6)	93.9 (±1.4)	(1.5 ± 0.3) × 10^3^	14.2 (±1.0)

**Table 2:** Color coordinates L^*^, a^*^, and b^*^ measured on WM and EAM mock-ups, without and with soiling mixture, and after cleaning with SA and KG gel by repeating applications. The average values and the related standard deviations were achieved by repeating the measurement five times. Standard deviation values are given in brackets. Wo.S.M. = without soiling mixture, W.S.M. = with soiling mixture.

Condition		SA Gel			KG Gel		
		L^*^	a^*^	b^*^	L^*^	a^*^	b^*^
**WM**	**Wo.S.M.**	75.8 (±0.6)	6.8 (±0.4)	39.7 (±0.7)	75.8 (±0.6)	6.8 (±0.4)	39.7 (±0.7)
	**W.S.M.**	68.5 (±1.9)	7.35 (±0.25)	35.7 (±0.9)	68.5 (±1.9)	7.35 (±0.25)	35.7 (±0.9)
	**0**	68.4 (±0.5)	8.8 (±0.4)	41.6 (±0.6)	70.7 (±0.7)	8.5 (±0.4)	41.2 (±0.5)
	**1**	71.6 (±0.6)	7.74 (±0.18)	41.2 (±0.6)	72.64 (±0.16)	7.38 (±0.19)	41.51 (±0.10)
	**2**	74 (±1)	6.53 (±0.22)	41.16 (±0.13)	74.00 (±0.25)	6.85 (±0.10)	41.37 (±0.12)
	**3**	72.0 (±0.7)	7.69 (±0.18)	41.6 (±0.3)	73.53 (±0.12)	7.20 (±0.02)	41.48 (±0.07)
**EAM**	**Wo.S.M.**	36.0 (±0.6)	4.9 (±0.3)	10.5 (±0.6)	36.0 (±0.6)	4.9 (±0.3)	10.5 (±0.6)
	**W.S.M.**	40.0 (±1.4)	3.4 (±0.6)	8.6 (±0.9)	40.0 (±1.4)	3.4 (±0.6)	8.6 (±0.9)
	**0**	35.6 (±0.6)	3.9 (±0.4)	9.2 (±0.5)	30.8 (±0.6)	4.6 (±0.3)	8.4 (±0.7)
	**1**	32.8 (±0.3)	3.8 (±0.3)	7.8 (±0.7)	33.1 (±2.5)	5.20 (±0.16)	9.9 (±0.9)
	**2**	35.2 (±1.0)	3.5 (±0.1)	7.9 (±0.3)	32.3 (±0.3)	5.1 (±0.3)	9.7 (±0.3)
	**3**	35.7 (±2.2)	3.87 (±0.18)	9 (±1)	32.0 (±2.3)	5.7 (±0.8)	10.2 (±1.7)

**Table 3:** The overall chromatic variation (△E^*^) calculated between the areas before and after cleaning the soiled-WM and soiled-EAM by repeating cleaning trials using SA and KG gels. Color variation between soiled-WM and WM is 8.3, and that between soiled-EAM and EAM is 4.7.

Condition		△E^*^	
		SA Gel	KG Gel
**WM**	**0**	6.1	6.0
	**1**	6.3	7.1
	**2**	7.8	7.9
	**3**	6.9	7.7
**EAM**	**0**	3.1	9.3
	**1**	7.3	7.2
	**2**	4.9	8.0
	**3**	4.3	8.5

**Table 4:** Normalized counts of Cl (Kα) on sweat-WM and sweat-EAM, before (control) and after different cleaning applications with SA and KG.

Mock-Up	Control	SA1	SA2	SA3	KG1	KG2	KG3
**WM**	11.94	0.11	0.09	0.09	0.10	0.12	0.12
**EAM**	3.35	2.50	1.50	0.52	1.90	1.12	0.49

## Data Availability

Data are contained within the article or supplementary materials.

## Supplementary Materials

The following supporting information can be downloaded at: https://www.mdpi.com/article/10.3390/ma15031100/s1, Table S1: Gelation characteristics of SA and KG; Figure S1: Images related to gelation of SA; Table S2: Reflection FTIR band assignment.
