# Supplementary material for: Preliminary Cleaning Approach with Alginate and Konjac Glucomannan Polysaccharide Gel for the Surfaces of East Asian and Western String Musical Instruments
Source: Materials (Basel). 2022 Jan 30;15(3):1100. doi: 10.3390/ma15031100 (PMC8838699; doi:10.3390/ma15031100)
Supplement: Supplementary file 1 [file materials-15-01100-s001.zip › materials-1555137-supplementary.pdf]

materials

Supplementary Materials

Preliminary Cleaning Approach with Alginate and Konjac Glucomannan Polysaccharide Gel for the Surfaces of East Asian and Western String Musical Instruments

Chaehoon Lee [1,2], Francesca Volpi [2,3], Giacomo Fiocco [2,*], Maduka L. Weththimuni [1], Maurizio Licchelli [1] and Marco Malagodi [2,3,*]

[1] Department of Chemistry, University of Pavia, 27100 Pavia, Italy
[2] Arvedi Laboratory of non-Invasive Diagnostics, CISRiC, University of Pavia, 26100 Cremona, Italy
[3] Department of Musicology and Cultural Heritage, University of Pavia, 26100 Cremona, Italy
* Correspondence: Correspondence: giacomo.fiocco@unipv.it (G.F.), marco.malagodi@unipv.it (M.M.); Tel.: +39-0372-567770

TABLE OF CONTENTS

- **Table S1: Gelation characteristics of SA and KG**
- **Figure S1: Images related to gelation of SA**
- **Table S2: Reflection FTIR band assignment**

Table S1. Gelation characteristics of SA with divalent and trivalent ions and KG with alkali and borax. pH value is the result by examining with pH strip.

Materials		pH	Characteristic of gelation and references
SA	$BaCl_2$	7	- As shown on Figure S1 (a), egg-shell structure, containing solvent inside has formed [1]
	$SrCl_2$	7	
	$CaCl_2$	7	- After forming a gel, a small amount of solvent inside diffused out of the shell over time, causing a leakage problem, which has already been reported in some research [2,3]<br>- SA in the form of film, crosslinked with 2% $CaCl_2$, reached the promised strength and flexibility for being used in cleaning tests as presented on Figure S1 (b) and (c). Also, in the research on the formation of alginate film, the results were shared that $CaCl_2$ at high concentrations of 3% and 6% was not suitable for the usage in biomedical applications, while 1.5% was recommended [4]
	$MgCl_2$	6	
	$Al_2(SO_4)_3$	4-5	- Alginate with aluminum, forms a three-dimensional bonding structure [5]. But the gel was breaking easily to be used for the cleaning system (Figure S1 (d))
KG	NaOH	12	- alkali and heat-induced gelation showed promising bonding of the gel [6], that can be used for cleaning<br>- the color of the gel was white on alkali-KG
	KOH	10-12	
	$Ca(OH)_2$	13	
	borax	7	- flexibility was enhanced as the concentration of borax and KG increased [7,8]. KG-borax gel is known not only for its flexibility but also for excellent self-healing properties [9,10]<br>- borax-KG gel was colorless and transparent

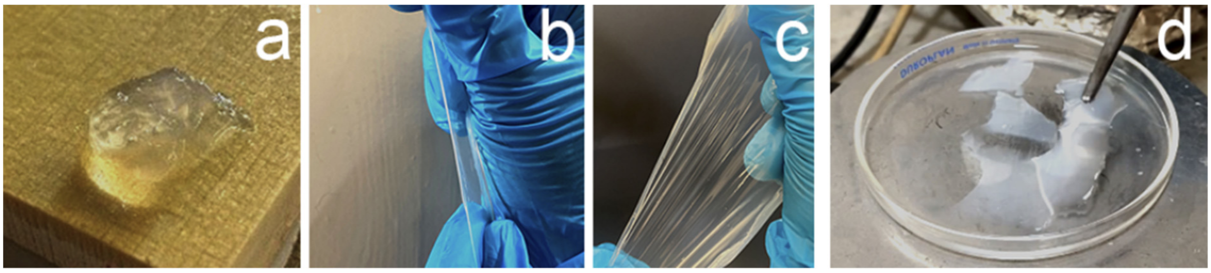

Figure S1. Gelation of SA with (a), (b) and (c) are gelation with Ca^{2+}. (a) formed in egg-shell structure, and after leaving for 10 min for cleaning, solvent diffused on the surface of WM. (b) and (c) show the film shape form and flexibility of SA gel by controlling the thickness in 2-3 mm. (d) SA showing easily breakable features with Al^{3+}.

Table S2. Reflection FTIR band assignment in the range 4000- 800 cm^{-1} identified on EAM and WM and on the soiling mixture and sweat dispersed on the mock-ups. For the derivative bands marked with an asterisk (*), the value refers to the maximum of the band after the application of KK transformations. Inv. = inverted band (Restrahlen) in the pseudo-absorbance spectra.

Attribution	Band assignment	Wavenumber (cm^{-1})	References in the manuscript
EAM (without depositions)	νC=O hemicellulose	1740	[61-63]
	νC=O lignin	1595	[61,63]
	aromatic skeletal vibration of lignin	1510	[61]
	aromatic CH deformation and $\nu_{as}CH_3$ in lignin	1460	[61]

	νC–O in lignin and xylan, syringyl ring	1240	[61,62,66]
WM (without depositions)	$\nu_{as}CH_3$, ν_sCH_3	2930-2860*	[12,63,66]
	C=O	1740-1730*	[63,66]
	δ_sCH_2, $\delta_{as}CH_3$, δ_sCH_3	1460*, 1380*	[63,66]
	νC-O	1250*, 1170*	[63,66]
Kaolin	νO-H	3620	[12,47,65-67]
	δSi-O	1020-1000	[12,47, 65-67]
	νO-H	915	[12,55,64-67]
Calcium carbonate	νC-O by calcium carbonation	1420, 875	[12,47]
Organic components	νC-H	2950-2825*	[12,66,68]
	νC-O	1650-1600*	[68]
Sweat	νC-H and νN-H	3200-3000*	[69-72]
	νC-N	1470-1410 (inv.)	[69-72]

Reference

1. Sikorski, P.; Mo, F.; Skjåk-Bræk, G.; Stokke, B.T. Evidence for egg-box-compatible interactions in calcium - Alginate gels from fiber x-ray diffraction. *Biomacromolecules* **2007**, *8*, 2098–2103, doi:10.1021/bm0701503.
2. Al-Musa, S.; Abu Fara, D.; Badwan, A.A. Evaluation of parameters involved in preparation and release of drug loaded in crosslinked matrices of alginate. *J. Control. Release* **1999**, *57*, 223–232, doi:10.1016/S0168-3659(98)00096-0.
3. Chuang, J.J.; Huang, Y.Y.; Lo, S.H.; Hsu, T.F.; Huang, W.Y.; Huang, S.L.; Lin, Y.S. Effects of pH on the Shape of Alginate Particles and Its Release Behavior. *Int. J. Polym. Sci.* **2017**, *2017*, doi:10.1155/2017/3902704.
4. Li, J.; Wu, Y.; He, J.; Huang, Y. A new insight to the effect of calcium concentration on gelation process and physical properties of alginate films. *J. Mater. Sci.* **2016**, *51*, 5791–5801, doi:10.1007/s10853-016-9880-0.
5. Brus, J.; Urbanova, M.; Czernek, J.; Pavelkova, M.; Kubova, K.; Vyslouzil, J.; Abbrent, S.; Konefal, R.; Horský, J.; Vetchy, D.; et al. Structure and Dynamics of Alginate Gels Cross-Linked by Polyvalent Ions Probed via Solid State NMR Spectroscopy. *Biomacromolecules* **2017**, *18*, 2478–2488, doi:10.1021/acs.biomac.7b00627.
6. Zhang, T.; de Vries, R.; Xu, X.; Xue, Y.; Xue, C. Microstructural changes during alkali- and heat induced gelation of konjac glucomannan. *Food Hydrocoll.* **2021**, *114*, 106552, doi:10.1016/j.foodhyd.2020.106552.
7. Gao, S.; Guo, J.; Wu, L.; Wang, S. Gelation of konjac glucomannan crosslinked by organic borate. *Carbohydr. Polym.* **2008**, *73*, 498–505, doi:10.1016/j.carbpol.2007.12.013.
8. Gao, S.; Guo, J.; Nishinari, K. Thermoreversible konjac glucomannan gel crosslinked by borax. *Carbohydr. Polym.* **2008**, *72*, 315–325, doi:10.1016/j.carbpol.2007.08.015.
9. Chen, Y.; Song, C.; Lv, Y.; Qian, X. Konjac glucomannan/kappa carrageenan interpenetrating network hydrogels with enhanced mechanical strength and excellent self-healing capability. *Polymer (Guildf).* **2019**, *184*, 121913, doi:10.1016/j.polymer.2019.121913.
10. Song, C.; Lv, Y.; Qian, K.; Chen, Y.; Qian, X. Preparation of konjac glucomannan–borax hydrogels with good self-healing property and pH-responsive behavior. *J. Polym. Res.* **2019**, *26*, doi:10.1007/s10965-019-1702-z.
